# Novel *ABCG5* and *ABCG8* Variants in Sitosterolemia: Insights Into Haemolysis, Calcium Dysregulation and Therapeutic Challenges

**DOI:** 10.1155/humu/9208468

**Published:** 2026-05-09

**Authors:** Prashant Warang, Rashmi Dongerdiye, Pradnya Dehadrai, Prachi Kamble, Neha Samanpalliwar, Manisha Madkaikar, Chandrakala Shanmukhaiah, Prabhakar S. Kedar

**Affiliations:** ^1^ Department of Haematogenetics, ICMR-National Institute of Immunohematology, King Edward Memorial (K.E.M.) Hospital Campus, Mumbai, India; ^2^ Department of Clinical Haematology, Seth GSMC and KEM Hospital, Mumbai, India

**Keywords:** *ABCG5*, *ABCG8*, calcium dysregulation, ezetimibe therapy, lipid metabolic disorder, plasma phytosterols, sitosterolemia

## Abstract

Sitosterolemia is a rare autosomal recessive lipid metabolic disorder caused by mutations in *ABCG5* or *ABCG8*, leading to pathological accumulation of dietary plant sterols. The condition is clinically heterogeneous, presenting with xanthomas, premature atherosclerosis and haematological abnormalities such as stomatocytosis, haemolytic anaemia and thrombocytopenia, making diagnosis particularly challenging. We investigated four patients with unexplained inherited haemolytic anaemia and a history of recurrent transfusions to identify molecular and functional mechanisms and explore therapeutic options. Haematological work‐up, including CBC, peripheral smear, reticulocyte count, EMA binding assay, red cell enzyme activity and HPLC for haemoglobinopathies, was performed, along with biochemical assays for liver, renal and lipid function. Plasma sterol levels were quantified using GC–MS, whereas functional assays included intracellular calcium flux under osmotic stress, red cell density profiling and ROS measurement. Whole‐exome sequencing identified homozygous nonsense variants: a novel *ABCG8* (p.Trp584∗) in two siblings, *ABCG5* (p.Arg243∗) in one patient and *ABCG8* (p.Ser129∗) in another. Two patients showed markedly elevated plasma phytosterols, predominantly stigmasterol and *β*‐sitosterol. Ezetimibe therapy (10 mg/day for 6 months) effectively reduced sterol levels but failed to normalise haemoglobin or significantly reduce overhydrated red cell populations. Functional studies demonstrated elevated intracellular Ca^2+^, enhanced influx under stress, increased ROS and a persistent population of stomatocytes. In silico prediction tools confirmed the pathogenicity of the identified variants. Persistent haemolysis despite sterol‐lowering therapy suggests irreversible red cell membrane damage due to sterol incorporation, altered fluidity, oxidative stress and calcium dysregulation. Our findings indicate that combining ezetimibe with Ca^2+^‐modulating therapy may represent a novel strategy to reduce haemolysis in sitosterolemia.

## 1. Introduction

Sitosterolemia, also known as phytosterolemia, is a rare autosomal recessive lipid metabolic disorder characterised by excessive intestinal absorption and reduced biliary excretion of plant sterols, leading to their pathological accumulation in blood and tissues [1]. The condition is caused by biallelic loss‐of‐function mutations in either the *ABCG5* or *ABCG8* genes, which encode sterolin‐1 and sterolin‐2, respectively—two ATP‐binding cassette (ABC) transporters that form a heterodimer responsible for sterol efflux in enterocytes and hepatocytes. This heterodimer plays a central role in maintaining sterol homeostasis by limiting dietary phytosterol absorption and promoting their biliary excretion [2, 3].

Clinically, sitosterolemia manifests with a wide spectrum of symptoms, including xanthomas, premature atherosclerosis and haematological abnormalities. Haematologic manifestations such as mild to chronic haemolytic anaemia, macrothrombocytopenia and stomatocytosis are significant yet often under‐recognised. Patients may present with fatigue, pallor, elevated reticulocyte counts and splenomegaly, with mild to severe anaemia, particularly in paediatric cases. The pathophysiology involves incorporation of phytosterols into erythrocyte membranes, leading to increased membrane fragility and red cell destruction [1, 4].

Although the first clinical descriptions of sitosterolemia were reported in 1974 based on elevated plasma plant sterols [5, 6], molecular confirmation of the disease—via identification of pathogenic variants in *ABCG5* or *ABCG8*—was only achieved in the early 2000s [7]. To date, approximately 110 individuals with molecularly confirmed sitosterolemia have been reported worldwide. However, the condition is likely underdiagnosed due to low clinical suspicion and limited access to appropriate biochemical and genetic testing [8].

Diagnosis is frequently delayed due to its clinical resemblance to familial hypercholesterolemia (FH) and the absence of plant sterol quantification in routine lipid profiles. Additionally, haematologic findings are often misattributed to other causes. Next‐generation sequencing (NGS) is currently the preferred method for definitive diagnosis, as plasma sterol analysis requires specialised instrumentation not commonly available in routine haematology laboratories.

In this study, we report four molecularly confirmed cases of sitosterolemia, mutations in either *ABCG5* or *ABCG8*, from Indian patients. We evaluated the effect of ezetimibe therapy (inhibitor of Niemann‐Pick C1‐like 1 [NPC1L1] intestinal sterol transporter) combined with a phytosterol‐restricted diet on their haematological, biochemical and plasma sterol parameters. Furthermore, we employed in silico functional analysis and molecular modeling to explore the structural and pathogenic implications of the identified variants. This is one of the few Indian series integrating molecular, biochemical and therapeutic findings in sitosterolemia and provides novel insights into its clinical and genetic spectrum.

## 2. Material and Methods

### 2.1. Patients′ Clinical Features and History

All four cases included in this study were referred to our centre for evaluation of unexplained haemolytic anaemia with a history of recurrent blood transfusions. Initial clinical and laboratory investigations were performed to exclude common causes of haemolysis, including haemoglobinopathies, autoimmune haemolytic anaemia, red cell membrane disorders and enzymopathies. Patients meeting these criteria subsequently underwent molecular analysis using whole‐exome sequencing (WES), and only those with confirmed pathogenic variants in *ABCG5* or *ABCG8* consistent with sitosterolemia were included in this case series.

### 2.2. Case Report

Case I is a 20‐year‐old female from Lucknow, Uttar Pradesh, India, born to nonconsanguineous parents, who presented with a long‐standing history of fatigue and failure to thrive. She had been symptomatic since the age of 10 years, primarily complaining of easy fatigability and a dragging‐type pain in the left upper quadrant of the abdomen. There was no history of gastrointestinal bleeding (haematemesis or melena), altered sensorium or abdominal distension. Her clinical history was notable for recurrent episodes of jaundice over the years, and the patient presented with severe anaemia (Hb 5.1 g/dL) associated with pallor and splenomegaly, but was haemodynamically stable. One unit of packed red blood cells (PRBCs) was administered in 2023, following which the haemoglobin increased to 7.0 g/dL with clinical improvement. No further transfusions were required as haemoglobin levels remained relatively stable despite ongoing haemolysis. Of note, her elder sister had undergone splenectomy for an undiagnosed haematological condition, raising the suspicion of a familial disorder. On evaluation, Case I was found to have anaemia and thrombocytopenia. Abdominal examination revealed significant splenomegaly measuring 19 cm. Notably, there was no evidence of portal hypertension or cirrhosis on imaging studies. Viral markers, including HIV, HBsAg and HCV, were negative.

Laboratory investigations showed elevated serum lactate dehydrogenase (LDH) levels at 438.6 U/L, consistent with ongoing haemolysis. Peripheral blood smear demonstrated hypochromia, anisocytosis and the presence of stomatocytes. Osmotic fragility testing was within normal limits. Both the Direct Coombs Test (DCT) and Indirect Coombs Test (ICT) were negative, making autoimmune haemolytic anaemia less likely; however, rare cases of Coombs‐negative AIHA have been described. High‐performance liquid chromatography (HPLC) revealed a normal haemoglobin pattern, excluding haemoglobinopathies such as sickle cell disease, *β*‐thalassemia and unstable haemoglobin variants.

In view of the chronic haemolytic anaemia with stomatocytosis, splenomegaly and negative Coombs and HPLC findings, a hereditary red blood cell (RBC) membrane disorder was strongly suspected. The absence of autoimmune or infectious causes, as well as normal haemoglobin studies, supported a nonimmune, nonhaemoglobinopathy aetiology. The patient was therefore referred to our centre for further evaluation and genetic testing to confirm the diagnosis and guide appropriate management.

Case II was a 16‐year‐old female sibling of Case I, born to nonconsanguineous parents. She has been symptomatic since the age of 6 years, presenting with chronic anaemia and splenomegaly. She has required blood transfusions on four occasions to date. Growth retardation (short stature) is noted, likely secondary to chronic haemolytic anaemia. On examination, splenomegaly was present. No dysmorphic features or other systemic abnormalities were reported. Laboratory investigations revealed a reticulocyte count of 10%, indicative of compensatory erythropoiesis. Peripheral blood smear showed stomatocytosis, macrocytosis and polychromasia, suggestive of ongoing haemolysis. The osmotic fragility test was normal. Laboratory investigations show negative DCT and ICT, haemoglobin electrophoresis was normal and tests for sickle cell disease, unstable haemoglobins and paroxysmal nocturnal haemoglobinuria (PNH) were negative. LDH was 566.3 IU/Hb.

The patient was referred for further evaluation of undiagnosed haemolytic anaemia. The findings, particularly stomatocytosis with normal osmotic fragility, raise the suspicion of a hereditary red cell membrane disorder, such as hereditary stomatocytosis (possibly dehydrated or overhydrated type). Genetic conditions involving *PIEZO1* or *KCNN4* mutations may be considered, warranting further genetic and membrane studies or Ion Channelopathies studies.

Case III was a 21‐year‐old female, born to consanguineous parents, who presented with a longstanding clinical history suggestive of a chronic haematological disorder. She exhibited features of anaemia and jaundice, accompanied by persistent fever, mild hepatomegaly and marked splenomegaly. Bone marrow examination revealed hypocellularity with evidence of erythroid dysplasia. She has received around 20 transfusions since early childhood. Peripheral blood smear demonstrated significant anisopoikilocytosis, polychromasia, occasional teardrop cells and elliptocytes. Given the clinical findings and transfusion history, differential diagnoses considered included inherited bone marrow failure syndromes (IBMFS), congenital dyserythropoietic anaemia (CDA), Diamond–Blackfan anaemia (DBA), congenital haemolytic anaemia (CHA) and red cell enzymopathies. The patient was subsequently evaluated using NGS molecular techniques to identify potential pathogenic variants contributing to her condition.

Case IV was a 27‐year‐old male born to consanguineous parents, who presented with anaemia and jaundice from early childhood, accompanied by splenomegaly. He is on regular blood transfusion support requiring an interval of 2–3 months. There is no significant family history present.

All patients underwent routine abdominal ultrasonography during their clinical evaluation. None of the patients demonstrated evidence of gallstones or cholelithiasis. In addition, none of the patients had clinical symptoms suggestive of premature atherosclerotic disease based on routine clinical evaluation; however, no dedicated cardiovascular assessment (e.g., vascular imaging or formal risk evaluation) was performed, and, therefore, subclinical atherosclerosis cannot be excluded.

None of the patients in our cohort demonstrated cutaneous or tendon xanthomas on clinical examination. The absence of xanthomas in our patients highlights the phenotypic heterogeneity of sitosterolemia, where some individuals may primarily present with haematological manifestations such as haemolytic anaemia and thrombocytopenia rather than classical lipid‐related features.

### 2.3. Haematological Laboratory Assays

Peripheral blood samples were collected in K₂‐EDTA, heparin and plain vacutainers following informed consent. The study was approved by the Ethics Committee of the ICMR–National Institute of Immunohaematology (Project Reference No: ICMR/NIIH/IEC/09/2024). Complete blood counts and haematological indices were measured using the Sysmex K‐1000 automated cell counter (Sysmex Corporation, Kobe, Japan). Haemoglobin variant analysis was performed by HPLC using the VARIANT Haemoglobin Testing System (Bio‐Rad Laboratories, Irvine, CA, United States).

#### 2.3.1. Biochemical Analysis

Liver function test, renal function test and lipid profile were done using a biochemistry analyser. Plasma sterol levels were quantified using GC–MS (gas chromatography‐mass spectrometry). Oxidative stress parameters were assessed as follows: Reactive oxygen species (ROS) levels were measured using 2 ^′^,7 ^′^‐dichlorofluorescein diacetate (DCF‐DA) [9]. To assess RBC membrane integrity, eosin‐5 ^′^‐maleimide (EMA) binding was analysed by flow cytometry [10]. Red cell enzyme activities—including glucose‐6‐phosphate dehydrogenase (*G6PD*), pyruvate kinase (PK), glucose phosphate isomerase (*GPI*) and pyrimidine‐5 ^′^‐nucleotidase (*P5* ^′^ *N*)—were determined using standard spectrophotometric methods [11].

#### 2.3.2. Functional Assays

Functional assays were performed using RBCs obtained from healthy control individuals (*n* = 25) with no history of haematological disorders. Control subjects were selected to be age and sex matched as closely as possible to the patient cohort. All experiments were performed with three replicates per sample, and each assay was repeated in at least two independent experimental runs to ensure reproducibility.

1. **Flow cytometric analysis of intracellular free Ca** ^**2+**^ **levels and swelling assay:** A whole heparinised blood sample in RBC plasma‐like buffer and calcium chloride (2 mM) was incubated with Fluo‐4 AM (Invitrogen) at a final concentration of 1 *μ*g/mL. After incubation in the dark for 1 h at room temperature, the fluorescent intensity was determined for 10,000 events in the FL‐1 channel using a Beckmann Coulter DxFlex flow cytometer at excitation 488 nm and emission 520 nm. The samples with similar preparation were spiked with 250‐*μ*L distilled water in order to check its effect on the activation of calcium channels, under swelling test.
2. **Flow cytometric analysis of ROS**: RBCs collected from subjects were washed three times with phosphate‐buffered saline (PBS) to prepare packed RBCs. RBC suspension (1 × 10^6^/mL) in PBS was incubated with 2 ^′^,7 ^′^‐DCFH‐DA (Sigma‐Aldrich, St. Louis, MO, United States) at a final concentration of 0.4 mM. After incubation at 37°C for 15 min in a humidified atmosphere of 5% CO_2_, RBCs were washed and resuspended in PBS. Fluorescence intensity was determined for 10,000 events in the FL‐1 channel using a Beckmann Coulter DxFlex flow cytometer at excitation 488 nm and emission 520 nm.
3. **Percoll density gradient assay**: Red cell density distribution was studied using the Percoll gradient separation method. Whole heparinised blood was used for this assay. Samples were mixed with RBC plasma‐like buffer and Percoll (density 1.13 g/mL) and subjected to ultracentrifugation to separate erythrocytes based on density. Ultracentrifugation was performed at 20,000 × g for 30 min at 37°C. Following centrifugation, distinct fractions corresponding to RBCs with different densities were obtained, allowing assessment of red cell density distribution [12, 13].

#### 2.3.3. DNA Extraction and Library Preparation for NGS

Genomic DNA was isolated from peripheral blood leukocytes using the FlexiGene DNA Kit (Qiagen, United States) and quantified with a Qubit fluorometer. Library preparation was performed using the Twist Library Preparation Kit 2.0 (Twist Biosciences).

#### 2.3.4. Bioinformatic Analysis

WES was performed, and variants were filtered based on quality and relevance to the clinical phenotype. Annotation was carried out using publicly available databases such as gnomAD for population frequency, ClinVar/HGMD/LOVD for previously reported variants and in silico prediction tools including CADD (PHRED score), MutationTaster, phyloP, phastCons and GERP to assess conservation and functional impact. Variants were classified according to the ACMG/AMP guidelines [14].

#### 2.3.5. Statistical Analysis

Statistical analysis was performed using GraphPad Prism (version X). Given the small sample size, most results were analysed descriptively and presented as mean ± SD or median values. Where appropriate, comparisons between patient samples and healthy controls were performed using the Mann–Whitney *U* test. A *p* value <0.05 was considered statistically significant.

## 3. Result

The clinical, haematological, biochemical and molecular data of all four patients at the time of study are summarised in Table 1. RBC morphology shows the presence of stomatocytes, polychromic macrocytes and thrombocytopenia (Figure 1). In this study, thrombocytopenia was transient and self‐limiting, with no associated clinical manifestations, and is therefore considered likely incidental rather than a characteristic feature of the disease. All three patients presented with mild to moderate anaemia (Hb range: 8.0–12.3 g/dL). All patients had a history of receiving blood transfusions. To rule out membrane and enzymatic defects, red cell membrane protein analysis using EMA dye‐based flow cytometry was performed, suggesting the absence of red cell membrane protein defect. Red cell enzyme activity assays for G6PD, PK and GPI were conducted using spectrophotometry and found to be within the normal range. HPLC analysis for haemoglobin revealed the absence of haemoglobinopathies (Table 1).

**TABLE 1:** Clinical, haematological, biochemical and molecular data at the time of investigations.

Parameters	Case I	Case II	Case III	Case IV	Normal range
Age/sex	20 years/F	16 years/F	21 years/F	27 years/M	
Origin	Lucknow, Uttar Pradesh	Lucknow, Uttar Pradesh	Bihar	Maharashtra	
Consanguinity	No	No	Yes	Yes	
Blood transfusion	1	4	Every 3–4 months	Every 3–4 months	
Feature	Splenomegaly	Splenomegaly	Mild hepatomegaly, gross splenomegaly	Splenomegaly	
**Haematological**					
RBC (×10^6/^mL)	2.69	2.67	3.01	4.78	M:4.5–5.5, F:3.8–4.8
HB (g/dL)	8.0	8.1	10.9	12.3	M:13–17.0, F:12–16
HCT (%)	27.1	29.6	36	37.3	38.8–50
MCV (fL)	100.7	110.9	119	78	80–100
MCH (pg)	29.7	30.3	36.2	25.7	27–32
MCHC (g/dL)	29.5	27.4	30.3	33	32–35
RDW (%)	17.1	18.6	14.8	15.1	11.6–14
WBC (×10^9^/L)	6.45	5.61	4.94	8.6	4–10
Platelets (×10^9^/L)	128	103	296	14	150–400
Reticulocyte (%)	3	10	20	1	< 2.0
Serum lactate dehydrogenase (U/L)	438.6	566.3	N/A	N/A	140–280
**Biochemical**					
EMA test (MCF)	77,260.84	79,258.5	83,748	84,598	76,300–85,700
G6PD (IU/g Hb)	8.90	9.500	11.77	10.70	6.5–13
PK (IU/g Hb)	8.80	9.81	12.65	10.47	9–14
GPI (IU/g Hb)	54.20	59.85	46.34	70.60	45–75
P5 ^′^N (ratio)	2.85	2.70	2.6	2.81	2.5–4.0
HbA_2_ (%)	2.3	2.4	2.7	2.4	1.5–3.5
Hb F (%)	0.4	0.3	1.5	1.5	< 2.0
**Molecular**					
	**Gene**	**Exon**	**DNA**	**Amino acid change**	**Zyg**
Case II	*ABCG8*	Exon 11	c.1751G > A	p.Trp584∗	Homo
Case II	*ABCG8*	Exon 11	c.1751G > A	p.Trp584∗	Homo
Case III	*ABCG5*	Exon 6	c.727C > T	p.Arg243∗	Homo
Case IV	*ABCG8*	Exon 4	c.386C > A	p. Ser129∗	Homo

FIGURE 1Peripheral blood smear of the patient (a) before treatment and (b) after treatment with ezetimibe (10 mg/day) for the indicated duration, showing morphological changes associated with therapeutic response.

**Figure:**
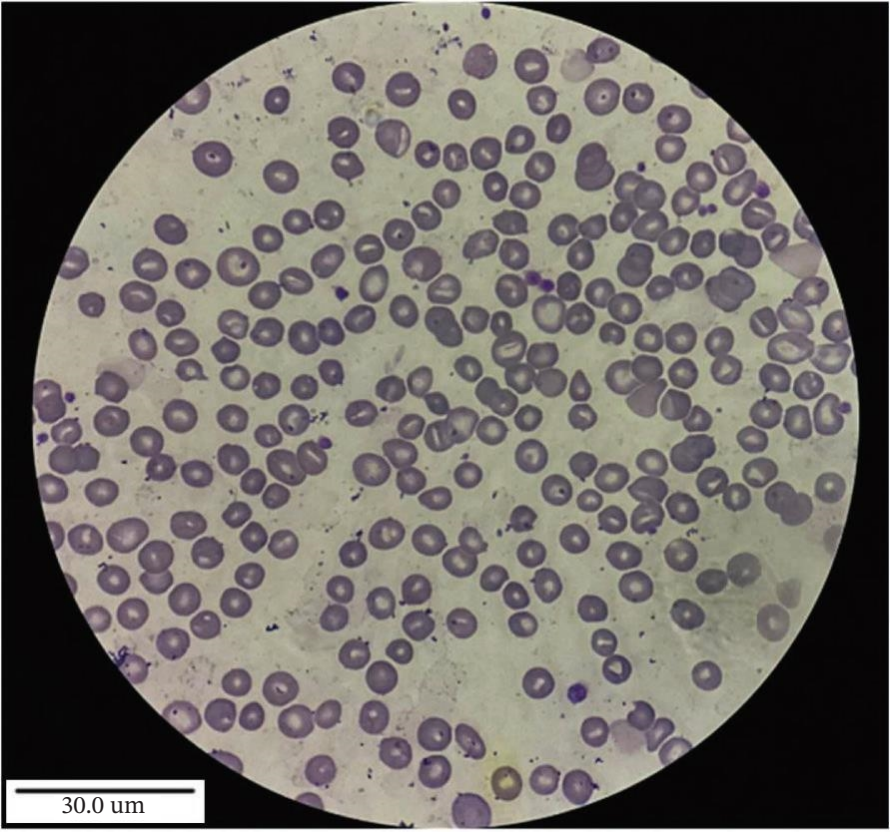
(a)

**Figure:**
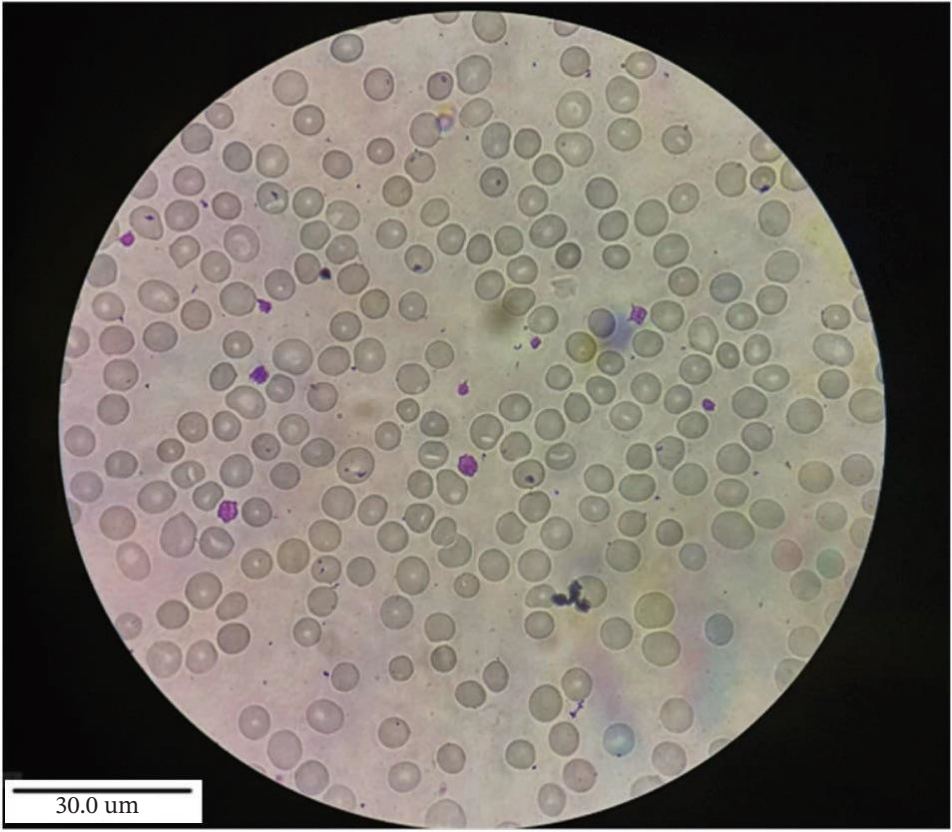
(b)

### 3.1. Bioinformatic Analysis

WES identified three nonsense variants in the *ABCG5* and *ABCG8* genes in cases with sitosterolemia. Two variants in *ABCG8* (p.Trp584∗ in Cases I and II and p.Ser129∗ in Case IV) and in Case III, p.Arg243∗ in the *ABCG5* gene, were detected, all of which are in a homozygous state. The *ABCG8* p.Trp584∗ variant was novel, whereas the other two variants had been previously reported in association with sitosterolemia. In silico prediction tools (CADD, MutationTaster, phyloP, phastCons, GERP) supported the pathogenic nature of these variants, consistent with ACMG classification as pathogenic/likely pathogenic (Table 2).

**TABLE 2:** Identified variants in *ABCG5* and *ABCG8* genes with in silico pathogenicity predictions.

Gene	Nucleotide change	Protein change	Location	Zygosity	Disorder	Consequence	Population frequency	ACMG	ClinVar/HGMD/LOVD	CADD (PHRED)	Mutation taster	PhyloP	PhastCons	GERP
ABCG8	c.1751G > A	p.Trp584∗	Exon 11	Homozygous	Sitosterolemia 1	Nonsense	Variant absent	PM2 and PVS1 (likely pathogenic)	Novel	51	1	4.286	1	1.68
ABCG5	c.727C > T	p. Arg243∗	Exon 6	Homozygous	Sitosterolemia 2	Nonsense	< 0.01%	PM2, PVS1 and PP5 (pathogenic)	Reported	41	1	3.995	0.998	4.68
ABCG8	c.386C > A	p.Ser129∗	Exon 4	Homozygous	Sitosterolemia 1	Nonsense	< 0.01%	PM2, PVS1 and PM3 (pathogenic)	Reported	38	1	1.331	0.941	4.03

### 3.2. Biochemical, Phytosterol and Haematological Response to Ezetimibe Therapy

Cases I and II received ezetimibe therapy at a dose of 10 mg/day for 6 months, whereas Cases III and IV were lost to follow‐up and therefore could not be evaluated further. The effect of ezetimibe on biochemical parameters, plasma phytosterol concentrations and haematological indices is summarised in Table 3. In both treated patients (Cases I and II), intracellular free calcium levels were significantly elevated compared to healthy controls (*p* = 0.05). Furthermore, these patients demonstrated an increased calcium influx in response to osmotic swelling (*p* = 0.05) and higher levels of ROS (*p* = 0.07), highlighting ongoing red cell dysfunction despite therapy, as illustrated in Figure 2.

**TABLE 3:** The effect of ezetimibe on biochemical parameters, plasma phytosterol concentrations and haematological indices.

		Case I		Case II	
		Before	After treatment	Before	After treatment
LFT	T bilirubin (mg/dL)	1.6	1	1.8	1.8
	Direct bilirubin (mg/dL)	0.8	0.4	1	0.8
	Indirect bilirubin (mg/dL)	0.8	0.6	0.8	1
	**SGOT (IU/L)**	17	17	34	24
	**SGPT (IU/L)**	16	14	46	25
	**GGPT(U/L)**	10	9	20	15
	**Alkaline phosphatase (IU/L)**	49	45	128	98
	Total protein (gm%)	8.1	7.7	8.1	7.4
	Albumin (gm%)	5.2	5.1	4.9	4.4
	Globulin (gm%)	2.9	2.6	3.2	3
	A:G ratio	1.793	1.961	1.53	1.466
RFT	Sr. calcium (mg/dL)	9.3	9	9.4	8.7
	Sr. phosphorus (mg/dL)	3.6	3.8	4.4	4.1
	Sodium (mMol/L)	134	139	138	140
	Potassium (mMol/L)	3.4	5.2	3.5	5.1
	Chlorides (mMol/L)	95	103	100	102
	HCO_3_(TCO_2_) (mMol/L)	26.2	22.8	22.9	23.2
	Anion gap	16.2	18.4	18.6	19.9
	Sr. uric acid (mgm%)	5.4	4.5	6.6	7
	Sr. creatinine (mgm%)	0.39	0.39	0.34	0.36
Lipid profile	**Cholesterol (mgm/dL)**	103	92	101	75
	Triglycerides (mgm/dL)	167	148	101	149
	**HDL cholesterol (mgm/dL)**	41	34	40	26
	**LDL cholesterol (calculated in mgm/dL)**	28.6	28.4	40.8	19.2
	Cholesterol/HDL ratio	2.51	2.71	2.53	2.88
	VLDL cholesterol (mgm/dL)	33.4	29.6	20.2	29.8
Sterol in plasma	Lanosterol (*μ*mol/L)	0.02	0.23	0.07	0.05
	Cholestanol (*μ*mol/L)	0.3	0.71	0.22	0.48
	Lathosterol (*μ*mol/L)	0.08	0.09	0.08	0.08
	7 DHC (*μ*mol/L)	0.11	0.05	0.13	0.36
	Desmosterol (*μ*mol/L)	0.21	0.92	0.29	1.03
	Cholesterol (*μ*mol/L)	2663.91	2379.41	2612.18	1939.74

Phytosterol	**Stigmasterol (*μ*mol/L)**	41.83	39.32	36.75	30.74
	Beta‐sitosterol (*μ*mol/L)	19.43	15.55	17.04	13.56
	7 DHC/cholesterol	0.000081	0.00003	0.000098	0.000038
	Lathosterol/cholesterol	0.00003	0.000042	0.000031	0.000043
	Cholestanol/cholesterol	0.000114	0.000301	0.000115	0.000249
	C‐reactive protein (mg/L)	0.3	0.2	3.7	0.9
Hematology					
	WBC (×10^3^/mL)	6.45	5.5	5.61	5.1
	RBC (×10^6^/mL)	2.69	2.75	2.67	2.24
	HB (g/dL)	8	7.9	8.1	6.3
	HCT (%)	27.1	25.1	29.6	23.2
	MCV (fL)	100.7	91.3	110.9	103.6
	MCH (pg)	29.7	28.7	30.3	28.1
	MCHC (g/dL)	29.5	31.5	27.4	27.2
	PLT (×10^9^/L)	128	114	103	61
	RDW (%)	17.1	15.8	18.6	22

FIGURE 2(a) Determination of intracellular free calcium levels and (b) assessment of changes in intracellular calcium in response to mechanical stimulation (distilled water–induced osmotic swelling) using the Fluo‐4‐AM fluorescence dye, (c) along with measurement of ROS levels before and after treatment with ezetimibe.

**Figure:**
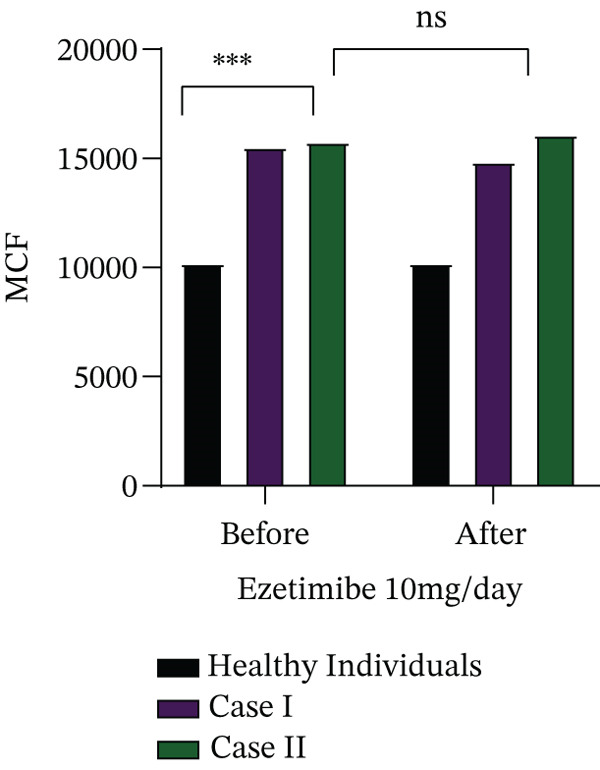
(a)

**Figure:**
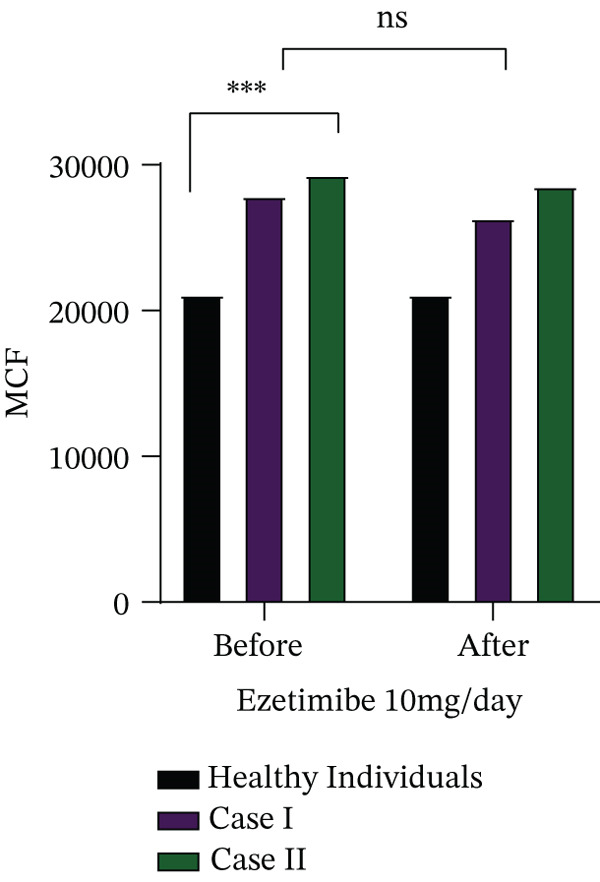
(b)

**Figure:**
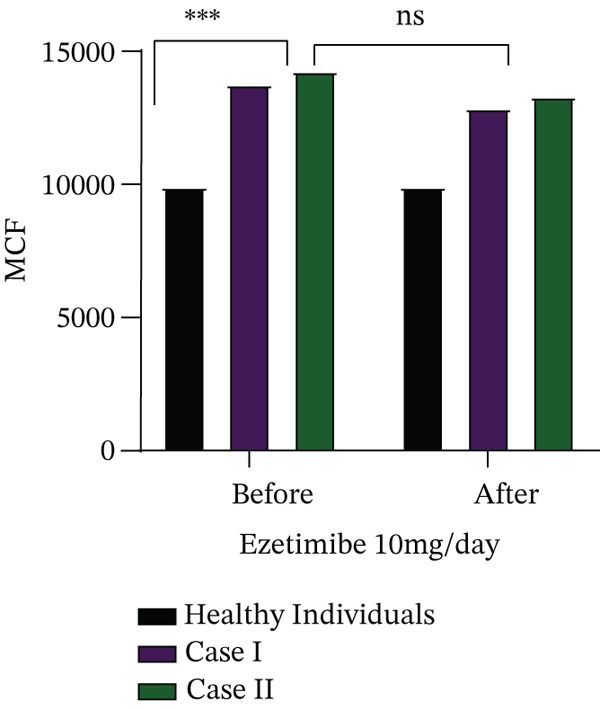
(c)

**FIGURE 3:**
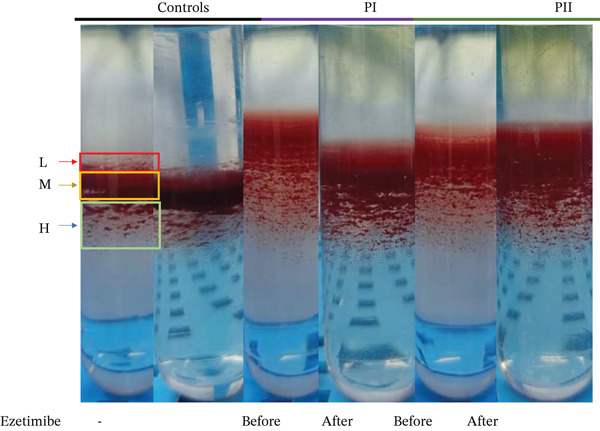
Red cell density distribution analysed using the Percoll gradient separation method before treatment and after treatment.

To further characterise red cell abnormalities, density distribution analysis was performed using Percoll gradient separation (Figure 3). Both patients exhibited a marked shift toward the low‐density red cell fraction, consistent with an overhydrated and osmotically fragile red cell population typical of stomatocytosis. Importantly, after 6 months of ezetimibe treatment, only minimal improvement was observed in the proportion of overhydrated cells, suggesting that sterol‐lowering therapy alone was insufficient to correct the underlying membrane defect. These findings indicate that while ezetimibe effectively reduces circulating phytosterol levels, it does not reverse the structural or functional red cell abnormalities associated with sterol incorporation into membranes. Persistent disturbances in calcium homeostasis, oxidative stress and abnormal red cell hydration may therefore contribute to ongoing haemolysis despite therapy.

## 4. Discussion

Sitosterolemia is a rare and underdiagnosed metabolic disorder that often mimics more common conditions such as FH or hereditary haemolytic anaemia. Interestingly, in our cohort, all patients presented with haemolytic anaemia and thrombocytopenia, yet had normal lipid profiles, a finding that challenges the conventional diagnostic assumption that hypercholesterolaemia is always present. Additionally, renal and hepatic function tests were within normal limits, except for mildly elevated bilirubin, consistent with ongoing haemolysis. In silico prediction tools, including MutationTaster and CADD, suggested that the identified novel *ABCG8* p.Trp584∗ variant is deleterious. Conservation analysis using phyloP, phastCons and GERP further demonstrated that the altered residue lies within a highly conserved region, underscoring its functional significance. The nonsense nature of the variant is consistent with loss of function, and ACMG criteria (PVS1 and PM2) support its classification as likely pathogenic. Taken together, these findings align with the clinical presentation and strengthen the pathogenic role of this variant.

Functional assays revealed novel insights into disease pathophysiology. Intracellular calcium levels, both at rest and under osmotic stress, were significantly elevated compared to controls, suggesting a role of calcium‐mediated membrane instability in red cell destruction. We also noted increased oxidative stress markers, including ROS, further contributing to red cell damage. In Cases I and II, Percoll gradient separation demonstrated overhydrated red cells, a hallmark of stomatocytic phenotypes. In Case II, the observed decrease in platelet count was transient and occurred in the context of the underlying disease, which is known to be associated with macrothrombocytopenia in sitosterolemia. The transient and asymptomatic nature of thrombocytopenia observed in this case indicates that it is unlikely to be a defining feature of the disease and may instead reflect an incidental variation.

The primary treatment of sitosterolemia is to reduce the phytosterols, mainly campesterol, stigmasteryl and beta‐sitosterol levels in the blood. Ezetimibe inhibits cholesterol absorption by binding to NPC1L1, a transmembrane protein in the epithelium of the small intestine, thereby reducing the level of cholesterol and related phytosterols in the blood. We observed that, despite 6 months of ezetimibe therapy (10 mg/day) and restriction on diet, which effectively reduced plasma phytosterol levels, haemoglobin levels remained low in these patients. This finding supports the hypothesis that sterol‐lowering therapy alone may not fully reverse haemolysis, especially once irreversible membrane changes have occurred. Given the persistently elevated intracellular calcium in our patients, we propose that adjunct therapies targeting calcium regulation might be beneficial in improving tologic outcomes.

In India, Desai et al. [15] described a case series of four patients aged 11–29 years, who predominantly manifested with haemolytic anaemia and macrothrombocytopenia. Peripheral blood smears revealed stomatocytes and giant platelets, whereas classical lipid‐related features such as xanthomas were absent. Genetic testing confirmed both homozygous and compound heterozygous mutations in the *ABCG5* gene. One patient achieved complete haematological recovery with ezetimibe therapy, whereas the others remained stable on dietary management. Khurana et al. [16] reported two cases of chronic haemolytic anaemia associated with compound heterozygous variants in *ABCG5*, consistent with sitosterolemia Type 2; both patients continue to be monitored for dietary adherence, blood counts and early atherosclerotic changes. More recently, Deka et al. [17] report a rare case of sitosterolemia presenting without dyslipidemia, highlighting the heterogeneity of clinical manifestations. The patient exhibited haematological abnormalities consistent with sitosterolemia despite normal lipid levels, underscoring the diagnostic challenges. This case emphasises the importance of considering sitosterolemia in unexplained haemolytic or haematological disorders, even in the absence of dyslipidemia. Jiang et al. [18] described a novel *ABCG5* mutation in patients with sitosterolemia presenting primarily with haematological abnormalities. Through genetic analysis and functional studies, they confirmed the pathogenic role of the variant and its impact on sterol transport. The study broadens the spectrum of *ABCG5* mutations and reinforces the link between sitosterolemia and haematologic disease.

In our cohort, a detailed clinical review did not reveal any history of active bleeding, gastrointestinal blood loss or other overt causes of haemoglobin decline during the follow‐up period. Routine biochemical investigations, including liver and renal function tests, did not indicate systemic inflammation or organ dysfunction that could significantly influence haemoglobin levels. Furthermore, markers suggestive of ongoing haemolysis, such as elevated LDH and reticulocytosis, remained consistent with chronic haemolytic anaemia rather than secondary causes. Additional factors such as iron status and inflammatory markers could further refine the interpretation; however, these data were not uniformly available for all patients during the follow‐up period and therefore represent a limitation of the study.

Routine clinical lipid assays are unable to distinguish phytosterols from cholesterol. However, under physiological conditions, circulating cholesterol concentrations are several hundred‐fold higher than phytosterol levels. Even when phytosterols increase substantially in sitosterolemia, their absolute contribution to total cholesterol measurements may remain relatively modest. This biochemical characteristic may partly explain why routine lipid panels in our cohort did not show marked hypercholesterolemia despite significantly elevated phytosterol levels detected by GC–MS [19, 20, 21].

In our study of two cases, persistent haemolysis despite ezetimibe therapy suggests the presence of irreversible membrane changes in RBCs. The accumulation of phytosterols in RBC membranes disrupts lipid composition and fluidity, rendering cells more rigid and prone to damage. Elevated intracellular calcium levels, observed in our functional assays, likely activate calpain‐mediated proteolysis of cytoskeletal proteins, compromising membrane integrity. In parallel, increased oxidative stress, evident from elevated ROS, further destabilises the membrane through lipid damage and protein cross‐linking. Cloos et al. [22] demonstrated that RBCs from sitosterolemia patients show altered membrane lipid composition and disrupted lipid distribution, leading to reduced deformability. These structural abnormalities were linked to sterol incorporation into RBC membranes, contributing to haemolysis. The study provides mechanistic insights into how phytosterol accumulation drives red cell fragility in sitosterolemia. These combined effects lead to microvesicle shedding, loss of membrane surface area and the formation of morphologically abnormal, overhydrated RBCs that are rapidly cleared by the spleen. As mature RBCs lack repair mechanisms, these membrane alterations become irreversible, contributing to chronic haemolytic anaemia unresponsive to sterol‐lowering therapy alone. To the best of our knowledge, this is the first systematic study evaluating the effect of ezetimibe on haematological and biochemical parameters, as well as on intracellular calcium and red cell density.

This study has several limitations. First, two patients (Cases III and IV) were lost to follow‐up, which limited the longitudinal assessment of treatment response and functional parameters. Second, two of the analysed patients (Cases I and II) were siblings, which may introduce genetic relatedness that could influence phenotypic comparisons. Therefore, the functional and comparative findings presented here should be interpreted cautiously and considered exploratory. Larger studies involving unrelated patients and longer follow‐up will be required to confirm these observations.

In conclusion, our study demonstrates that sitosterolemia can present with severe haemolytic anaemia and normal lipid profiles, which may delay diagnosis. Two cases had preserved liver and kidney function except for isolated hyperbilirubinemia. Molecular confirmation and functional profiling revealed intracellular calcium dysregulation and oxidative stress as contributing factors to red cell destruction.

Although ezetimibe effectively reduced plasma phytosterol levels, improvement in haemoglobin was not observed during the study period and may be influenced by multiple clinical factors or due to irreversible red cell membrane alterations rather than treatment failure alone. Recent reports have also suggested that combination therapy, including a phytosterol‐restricted diet, ezetimibe and immunomodulatory agents (thalidomide, tofacitinib and/or transfer factor), may improve haematological manifestations in sitosterolemia. Immunomodulators may reduce inflammatory burden and oxidative stress, which could potentially mitigate red cell damage. This concept is consistent with our observations of increased intracellular Ca^2+^ levels and elevated ROS in patient erythrocytes. However, none of our patients received immunomodulatory therapy, and further studies are required to determine whether such approaches can improve anaemia in sitosterolemia [23].

Targeting calcium in sitosterolemia could involve agents like *PIEZO1* inhibitors or antioxidants that stabilise membranes and reduce secondary calcium overload. This combination (ezetimibe + Ca^2+^‐targeting strategy) could reduce both primary sterol‐induced membrane fragility and secondary Ca^2+^‐mediated haemolysis. This represents a novel therapeutic direction to address persistent haemolysis not corrected by ezetimibe alone.

## Author Contributions

Prabhakar S. Kedar and Prashant Warang: conceptualisation, data curation, funding acquisition, investigation, project administration, writing – review and editing. Rashmi Dongerdiye, Pradnya Dehadrai, Neha Samanpalliwar, and Prachi Kamble: laboratory data analysis, molecular experimental work, sequencing experiments and variant validation. Chandrakala Shanmukhaiah and Manisha Madkaikar: patients′ clinical data, clinical case investigations.

## Funding

This study was supported by the Department of Health Research (DHR), File No. R.12020/08/2024‐HR/E‐Office:8292872.

## Disclosure

All authors participated in the data discussion and read and approved the manuscript. All authors have read and agreed to the published version of the manuscript.

## Ethics Statement

The study has been carried out in agreement with the research rules of our institutional ethical committee on human testing. This study has been approved by the National Institute of Immunohematology Ethical Committee, and as per protocol, written informed consent was obtained from all the patients or their parents.

## Consent

Written informed consent was obtained for all participants in this study.

## Conflicts of Interest

The authors declare no conflicts of interest.

## Data Availability

The whole data are summarised in the manuscript. Data are available on request from the author.
